# Single-cell Raman spectroscopic analysis of bacteroids in soybean nodules to observe the relationship between biomolecular constituents and symbiotic nitrogen fixation activity

**DOI:** 10.5511/plantbiotechnology.25.0414a

**Published:** 2025-09-25

**Authors:** Shunnosuke Suwa, Masahiro Ando, Kohki Kashiwagi, Takuma Kyotani, Kento Hasegawa, Habibi Safiullah, Masako Kifushi, Yohei Nishikawa, Toyoaki Anai, Naoko Ohkama-Ohtsu, Haruko Takeyama

**Affiliations:** 1 Department of Advanced Science and Engineering, Graduate School of Advanced Science and Engineering, Waseda University, 3-4-1 Okubo, Shinjuku-ku, Tokyo 169-8555, Japan; 2 AIST-Waseda University Computational Bio Big Data Open Innovation Laboratory (CBBD-OIL), 3-4-1 Okubo, Shinjuku-ku, Tokyo 169-8555, Japan; 3 Research Organization for Nano and Life Innovation, Waseda University, 513 Wasedatsurumaki-cho, Shinjuku-ku, Tokyo 162-0041, Japan; 4 Department of Life Science and Medical Bioscience, Graduate School of Advanced Science and Engineering, Waseda University, 2-2 Wakamatsu-cho, Shinjuku-ku, Tokyo 162-8480, Japan; 5 Faculty of Agriculture, Tokyo University of Agriculture and Technology, 3-5-8 Saiwai-cho, Fuchu, Tokyo 183-8509, Japan; 6 Biomanufacturing and Process Research Center (BPRC), National Institute of Advanced Industrial Science and Technology (AIST), 1-1-1 Higashi, Tsukuba, Ibaraki 305-8566, Japan; 7 Faculty of Agriculture, Kyushu University, 744 Motooka, Nishi-ku, Fukuoka, Fukuoka 819-0395 Japan; 8 Institute of Agriculture, Tokyo University of Agriculture and Technology, 3-5-8 Saiwai-cho, Fuchu, Tokyo 183-8509, Japan; 9 Institute for Advanced Research of Biosystem Dynamics, Waseda Research Institute for Science and Engineering, Waseda University, 3-4-1 Okubo, Shinjuku-ku, Tokyo 169-8555, Japan

**Keywords:** bacteroid, machine learning, Raman spectroscopy, soybean, symbiotic nitrogen fixation

## Abstract

Nitrogen fixation in soybean occurs as a result of symbiosis between the plant and rhizobia in the nodules. This process allows both the plant and the symbiont to acquire vital nutrition. To fully understand the symbiosis, many researchers have attempted to attain a deeper interpretation of the biomolecular behavior or enhance the nitrogen fixation activity of bacteroids. However, most studies have focused on forward and reverse genetics approaches to evaluate the contribution of a particular gene/enzyme in nitrogen fixation. Few studies have observed the bacteroids’ overall biomolecular behavior in the nodules. Thus, we grew soybean plants and recorded acetylene reduction assay (ARA) results at several growth stages. Simultaneously, we analyzed the biomolecular compounds in the bacteroids in the nodules at the single-cell level by Raman microspectroscopy. Random forest regression, a machine learning method, was applied to discover the biomolecular contribution to the ARA, as it predicted ARA results with high accuracy. Polyhydroxybutyrate (PHB) biopolymer significantly contributed to predicting ARA results, suggesting its potential relevance in symbiotic nitrogen fixation in soybean. Further studies related to PHB behavior will lead to a deeper understanding of symbiotic nitrogen fixation and may help achieve better control of this process to increase crop yields.

## Introduction

Symbiotic nitrogen fixation allows leguminous plants such as soybeans, peas, or *Lupinus* to utilize atmospheric nitrogen (Liu et al. 2018). In such plants, rhizobia in the soil induce the formation of nodules on the root surface. Inside nodules, within organelle-like symbiosomes, rhizobia differentiate into bacteroids, which incorporate atmospheric nitrogen, convert it to ammonium, and supply it to the plant, simultaneously receiving carbon sources from it (Fonseca-García et al. 2021; Schulte et al. 2021). The plant cells utilize ammonium in biologically available forms such as glutamate or glutamine (Sulieman et al. 2024).

Although numerous studies have revealed the behavior of biomolecules in the leguminous symbiotic nitrogen fixation, this phenomenon is not completely understood. Many studies have focused on the genomic or enzymatic aspects of symbiosis, using transcriptome analysis and/or gene editing to observe the function of specific proteins (Chen et al. 2023; Fan et al. 2017; Hakoyama et al. 2009; Nishida et al. 2021; Pessi et al. 2007; Saini et al. 2022; Wang et al. 2019). For this purpose, a given strain is inoculated onto the plant and the fluctuations in plant mass, nodule size, number, and nitrogen fixation activity are evaluated. However, few studies have focused on the biomolecules in bacteroid cells in their symbiotic states in nodules. A better understanding of nitrogen fixation in this aspect would lead to more effective control of this process because not only enzymes but also other biomolecules such as metabolites may be involved, as suggested by transcriptome analyses (Chen et al. 2023; Wang et al. 2019).

Raman spectroscopy is a vibrational spectroscopic technique that provides molecular structural information through characteristic Raman bands in a non-destructive or minimally invasive manner (Das and Agrawal 2011; Pezzotti 2021). We have published a wide range of in situ microbial biomolecular analyses using Raman spectroscopy combined with multivariate analysis (multivariate curve resolution-alternating least squares, MCR-ALS). This approach allows a deeper understanding of the biomolecules in the cells, such as the localization of secondary metabolites (e.g., penicillin in *Penicillium chrysogenum* and avermectin in *Streptomyces avermitilis*) (Horii et al. 2020, 2023; Samuel et al. 2022). Additionally, integrating this spectroscopic method with machine learning algorithms has facilitated the prediction of growth phases in *Chaetoceros tenuissimus* by simultaneously detecting multiple metabolites, including fatty acids and saccharides (Ando et al. 2023). These successful applications suggest the potential utility of Raman spectroscopy for bacteroid biomolecular analysis.

Here, we identify biomolecular components affecting symbiotic nitrogen fixation by linking soybean nitrogen fixation activity to single-cell bacteroid biomolecular profiles from Raman spectroscopy. Key findings include the plant growth phase and nodule size-dependent fluctuations in the acetylene reduction assay (ARA), molecular diversity in bacteroids, and the prediction of symbiotic nitrogen fixation activity using random forest regression (RFR). Polyhydroxybutyrate (PHB) was found to contribute to soybean’s symbiotic nitrogen fixation. PHB has previously attracted attention mainly as a biopolymer or energy source (Ratcliff et al. 2008; Trainer and Charles 2006). This discovery will open up new avenues for a better understanding of symbiotic nitrogen fixation, enhancing crop production.

## Materials and methods

### Plant material

Soybean (*Glycine max*) cultivar ‘Fukuyutaka’ was grown in a custom-made chamber, with 10–12 h of light a day, a temperature of 25–32°C, and humidity between 40% and 60%, using pots of 12 cm in diameter and 11 cm in height. The soil (Gray lowland soil) used for the experiment was taken from the soybean fields of Saga University (33°14′35.0″N 130°17′25.0″E). In total, the experiment comprised 12 pots of soybean plants. For each pot, two holes (2 cm in diameter and 8 cm in height) were dug and two seeds were sown in each. After germination, plants in each hole were thinned, leaving only the larger plant.

### ARA measurements

The ARA allows for the estimation of the nitrogen fixation activity. The measurement followed a published protocol (Mortuza et al. 2020). The amount of ethylene produced by the nitrogenase activity on acetylene was determined. The measurements and subsequent Raman spectroscopic observation were conducted at 30, 45, and 87 days, corresponding to the growth stages of nodulation (Nod), flowering (Flo), and ripening (Rip). Four pots were used as replicates each day. Plants were removed from the pots and soil. The root part of the plants was washed with tap water. Subsequently, nodules were collected from the roots. Moisture was provided to prevent the nodules from drying. Samples were divided into three groups because studies have revealed that legume nodules’ nitrogen fixation activity varies according to size (King and Purcell 2001; Tajima et al. 2007). Groups 1, 2, and 3 (G1, G2, and G3, respectively) refer to the nodules with diameters <1.5 mm, 1.5–2.0 mm, and >2.0 mm. For each group, 15 nodules were collected from one pot and used for the ARA measurement. The sum of these 15 nodules’ ARA results is the total ARA value in each condition. The nodule weight was also recorded and used to calculate the value per weight.

### Sample preparation and Raman measurements

Nodules for Raman spectroscopic measurements were sterilized by immersing them in 1% sodium hypochlorite solution for 1 min and then in 70% EtOH for 1 min. Next, nodules were washed by immersing them in distilled water for 1 min, twice. Subsequently, bacteroids were extracted according to the procedure in Supplementary Figure S1. Cover glass (18×18 mm, Matsunami Glass Ind., Ltd., Osaka, Japan) was put on top of the bacteroid cell suspension on a bigger cover glass (NEO Cover Glass 24×50 mm, Matsunami Glass Ind., Ltd.) and the sample was placed on the instrument. The optical condition of the laboratory-built Raman spectrometer was the same as that in a previous study, except for the objective (100×, 1.30 NA) (Horii et al. 2023). The laser power was set to 10 mW and irradiation time was 1 s, measured 10 times continuously. Twenty-seven bacteroid samples from the nodules were analyzed, nine nodules from each plant growth phase. For each sample, 29–32 single bacteroid cells were analyzed. In the case of Raman imaging measurement, agarose of ultra-low gelling temperature (Sigma-Aldrich, MO, USA) was added at a final concentration of 1% (w/v) when placing the cover glass on top of the suspension to prevent the optical tweezer from moving and rotating the cell body. A portion of the bacteroid cell suspension was used for 16S rRNA gene sequencing to analyze bacterial diversity to confirm the dominance of rhizobia in the nodules.

### Bacterial diversity analysis

Microbial DNA was extracted using an Extrap Soil DNA Kit Plus ver.2 (BioDynamics Laboratory Inc., Tokyo, Japan). The V3–V4 hypervariable regions of 16S rRNA genes were analyzed according to the Illumina protocol for 16S metagenomic sequencing library preparation with 341F and 806R primers (forward, 5′-TCGTCGGCAGCGTCAGATGTGTATAAGAGACAGCCTACGGGNGGCWGCAG-3′; reverse, 5′-GTCTCGTGGGCTCGGAGATGTGTATAAGAGACAGGACTACHVGGGTATCTAATCC-3′). PCR was performed following the above-mentioned protocol with the following temperature profile: 30 cycles at 95°C for 30 s, 52°C for 30 s, 72°C for 45 s, and a final extension at 72°C for 5 min. Barcoded amplicons were sequenced using the Illumina MiSeq 2×300 bp platform with the MiSeq Reagent Kit v3 (Illumina Co., CA, USA), according to the manufacturer’s instructions.

Raw read quality control was performed following the protocol of the Bioengineering Lab. Co. Ltd. (https://gikenbio.com/), with slight modifications. Raw reads containing primer sequences were extracted and trimmed using Cutadapt v3.1 (Martin 2011). Pairs of R1 and R2 were reconstructed using repair.sh v38.86. Bases below Q20 were trimmed, and reads under 40 bp in length were removed using fastp v0.21.0 (Chen et al. 2018). Pair ends were merged using flash2 (Magoč and Salzberg 2011). Merged reads of less than 250 bp were removed using fastp v0.21.0. Merged paired-end sequence reads were processed using the dada2 plugin in QIIME 2 (ver. 2020.11) (Bolyen et al. 2019) for denoising, calling the amplicon sequence variant (ASV), and generating a feature table of the ASV count tables. Taxonomic assignment was performed using the q2-feature-classifier (Bokulich et al. 2018) and the classify-sklearn naiūve Bayes taxonomy classifier against the Silva 138 99% operational taxonomic unit (OTU) full-length sequences. ASVs were subjected to a homology search against the nt database, using BLAST+ v2.10.1 (Camacho et al. 2009), and eukaryotic ASVs were removed.

Downstream analyses were performed using the phyloseq bioconductor package (McMurdie and Holmes 2013) and nyankomicro (https://github.com/xvtyzn/nyankomicro (Accessed Nov 25, 2022)) in R v4.1.2.

### Data analysis

The recorded Raman spectra were preprocessed by a white light spectrum to calibrate the detector sensitivity and by an indene spectrum for wavenumber calibration, as described in a previous study (Horii et al. 2023). Singular-value decomposition and spectral reconstruction were performed for noise reduction (Ando and Hamaguchi 2013; Huang et al. 2012), using Igor Pro (WaveMetrics, OR, USA).

MCR-ALS was carried out for the preprocessed Raman spectra. As a result, the obtained Raman spectrum matrix (A) was decomposed into two matrices: A=WH+E, where W refers to the MCR spectral components; H, to the intensity profile corresponding to the spectral components; and E, to the residual. LASSO regularization of α*_L_*_1_*_H_*=5*e*−05 was applied to prevent overfitting (Ando and Hamaguchi 2013; Huang et al. 2012; Suwa et al. 2024). MCR analysis was performed using the SciPy library in Python.

Machine learning methods principal component analysis (PCA) and RFR were applied to the MCR resolved biomolecular profile H after standardization, using the aerial intensity of H_2_O Raman bands, fitting three Gaussian functions between 3150 and 3700 cm^−1^. Random forest regression was performed along with the optimization of the hyperparameters by grid search. These analyses were conducted using the scikit-learn library in Python.

## Results and discussion

### ARA measurement and nodule bacterial diversity analysis

**Figure 1:**
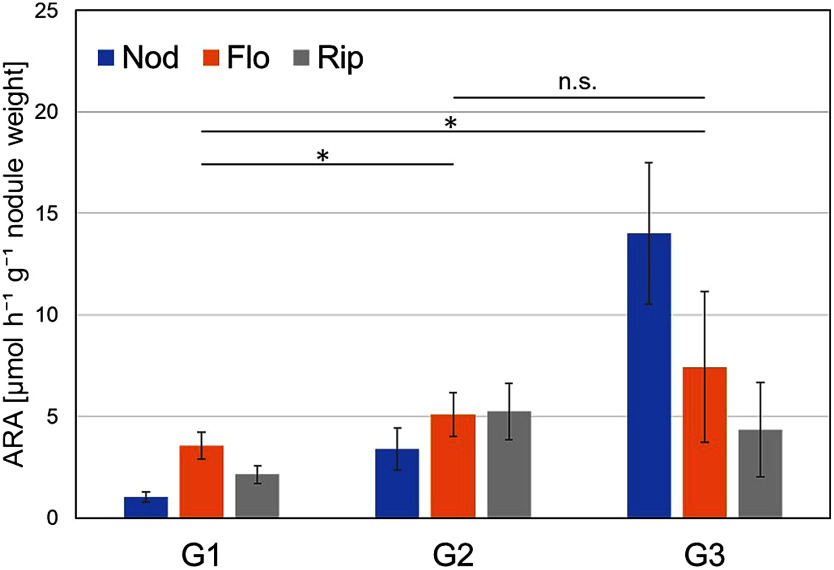
Figure 1. Recorded ARA results at each growth stage (Nod, Flo, and Rip) and nodule size (G1, G2, and G3). The error bars represent the standard error of four biological replicates per condition. Asterisks indicate statistically significant differences between nodule size groups (* *p*<0.05, *t*-test).

The ARA indicated that symbiotic nitrogen fixation activity depended on the plant growth phase and nodule size, as reported by previous studies (Figure 1, raw data in Supplementary Table S1) (Tajima et al. 2007). Larger nodules exhibited higher nitrogen fixation activity, as confirmed by *t*-test analysis (*p*<0.05). The plant growth phase also affected the ARA results. In the Nod phase, the results increased according to the nodule size, similar to the Flo phase. On the other hand, in the Rip phase, nitrogen fixation did not drastically differ regardless of nodule size. Although ARA outcome was diverse among growth conditions, the cross-sectional color of all nodules remained red or pink (representative images shown in Supplementary Figure S2), emphasizing the importance of quantitative methods for accurately assessing nitrogen fixation activity.

The bacterial diversity analysis using the 16S rRNA gene sequencing results from nodule extractions demonstrated the dominance of rhizobia, with *Bradyrhizobium* accounting for nearly 100% relative abundance across all 27 nodules. Thus, subsequent Raman spectroscopic analyses focused exclusively on rhizobia within the nodules, without interference from non-rhizobial bacteria outside the nodules (Supplementary Figure S3).

### Raman spectral components and single-cell imaging

**Figure 2:**
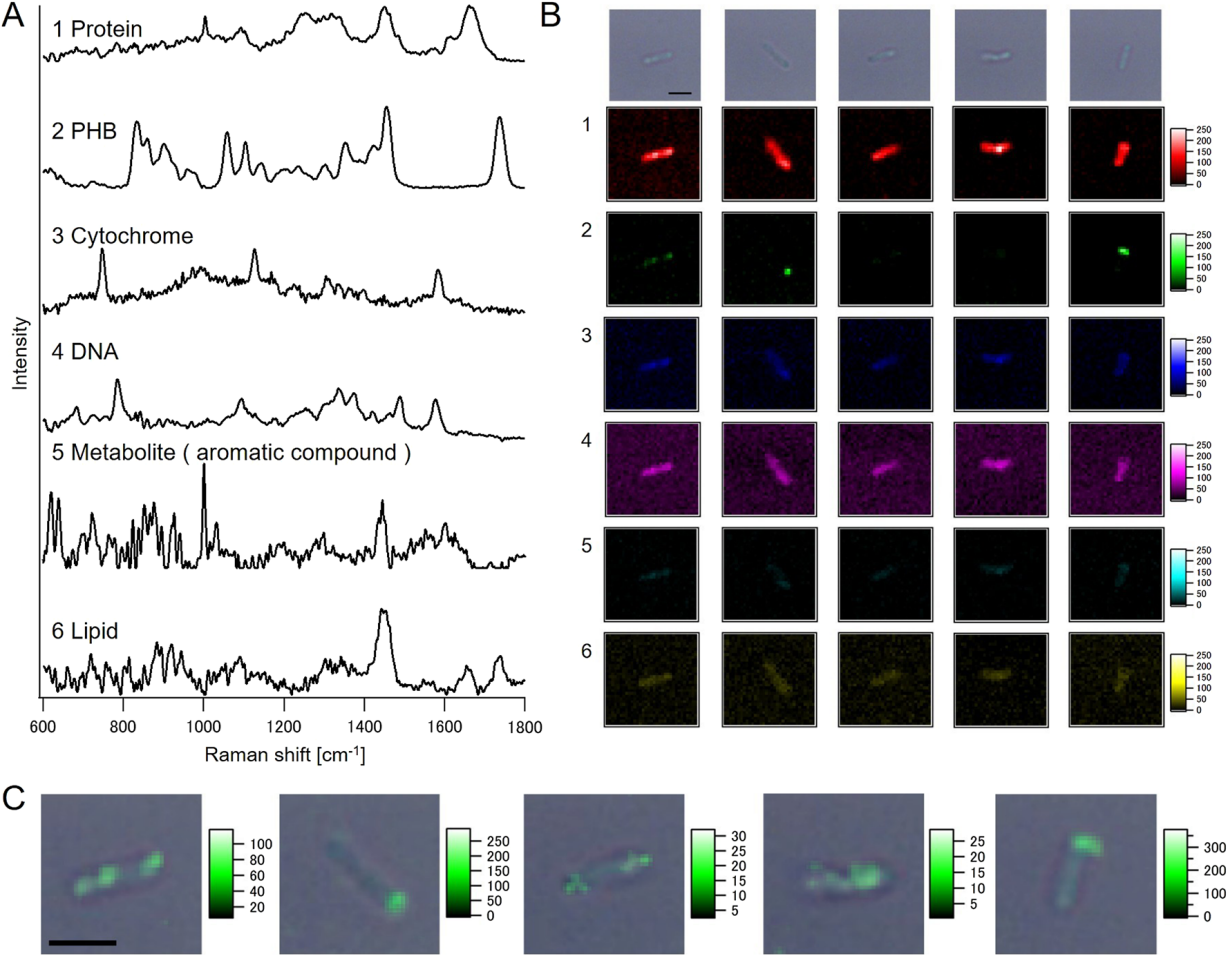
Figure 2. MCR resolved Raman spectral components detected in the bacteroids and Raman images. A) Raman spectra of (1) protein, (2) PHB, (3) cytochrome, (4) DNA, (5) metabolite (with a phenyl ring), and (6) lipid. B) Raman images of single-cell bacteroids in the Nod phase. Each component (1–6) corresponds to the Raman spectral constituents on the left. C) Overlay bright field and Raman images of PHB in an individual bacteroid cell. The color scales are modified in each image. The Raman images of PHB were smoothed via cubic polynomial interpolation with a 4×4 neighborhood value. Scale bar=2 µm.

Single-cell Raman spectroscopic observation of bacteroids across all plant growth phases (Nod, Flo, and Rip; 828 cells in total) and subsequent MCR-ALS analysis revealed the existence of several biomolecules in the cells (Figure 2A). Raman spectral component 1 represents proteins, indicated by the characteristic Raman bands at 1004 cm^−1^ (ring-breathing mode of phenylalanine and tryptophan residues), 1250 cm^−1^ (amide III), 1451 cm^−1^ (C-H bending), and 1661 cm^−1^ (amide I) (Huang et al. 2004; Rygula et al. 2013). Component 2 was identified as the PHB biopolymer by its characteristic Raman bands at 834, 902, 1058, 1104, 1352, 1455, and 1736 cm^−1^ (Hermelink et al. 2011; Tao et al. 2016). Component 3 was assigned to cytochrome, with major Raman bands at 747, 1126, 1304, 1332, and 1583 cm^−1^ (Kakita et al. 2012). Component 4 represents DNA, as indicated by Raman bands at 684, 785, 1095, 1364, 1374, 1488, and 1577 cm^−1^ (Falamas et al. 2013). Raman spectral component 5 showed a low signal-to-noise (S/N) ratio, yet showed characteristic bands at 1001 cm^−1^ and 1445 cm^−1^, which can be attributed to the ring-breathing mode of phenyl groups and CH_2_ deformation, respectively, suggesting metabolites of aromatic compounds. Component 6 can be assigned to a lipid with ester bonds by its characteristic Raman bands at 1091 cm^−1^ (C-C vibration), 1443 cm^−1^ (C-H_2_ deformation), 1655 cm^−1^ (C=C stretching vibration), and 1738 cm^−1^ (C=O vibration of the ester bond) (Czamara et al. 2015; Wu et al. 2011).

Single-cell Raman imaging of bacteroids from the Nod phase (Figure 2B) confirmed the presence of PHB in the form of granules in the cell (Figure 2C). Transmission electric microscopy (TEM) studies have reported that PHB is present as granules within cells (Giraud et al. 2013; Prakamhang et al. 2015; Quelas et al. 2016). Although these observations relied on the interpretation of the obtained image, hypothesizing that the white granules were PHB, Raman spectroscopy enabled molecular-based observation of this biopolymer and produced a result consistent with the mentioned TEM studies. Other cellular components were localized homogeneously in the cell.

### Biomolecular profile changes among different growth conditions

**Figure 3:**
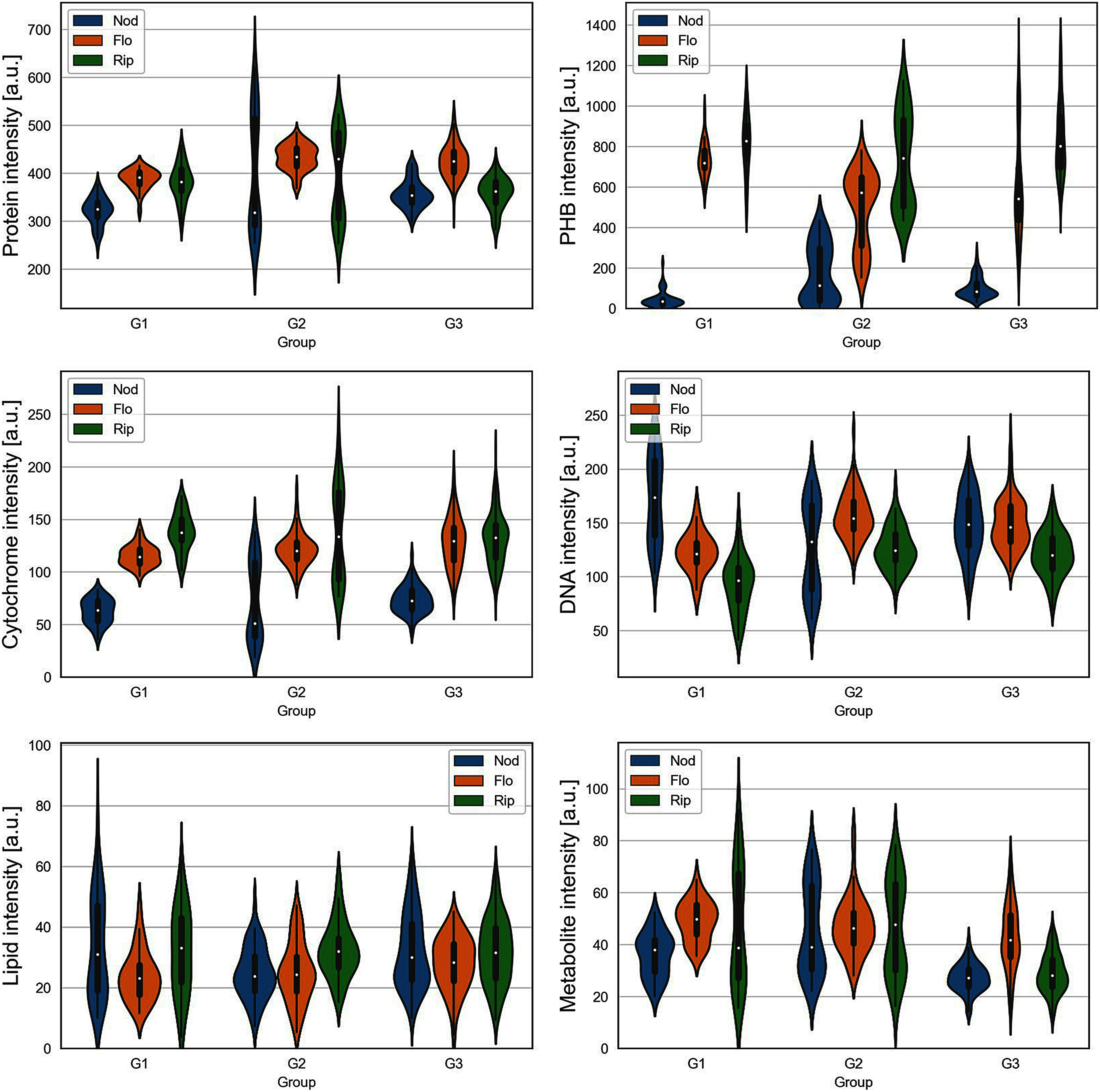
Figure 3. Violin plots for biomolecular components at each growth stage (Nod, Flo, and Glo) and nodule size (G1, G2, and G3).

Single-cell Raman spectroscopy revealed distinct changes in bacteroid biomolecular profiles across plant growth phases and nodule sizes (Figure 3). For this analysis, we examined a total of 828 individual bacteroid cells, with detailed cell counts for each condition provided in Supplementary Table S2. Although the protein content in bacteroid cells fluctuated across conditions, no consistent pattern emerged. By contrast, the PHB content increased progressively through the plant growth phases, with bacteroid cells in the Nod phase containing less PHB than those in the Flo and Rip phases. Cytochrome levels exhibited a similar trend, though less markedly than PHB. On the other hand, the DNA content in bacteroid cells slightly decreased with plant growth progression. Lipid and metabolite levels remained fairly stable during growth. Notably, nodule size also influenced the biomolecular profiles, particularly the PHB content.

**Figure 4:**
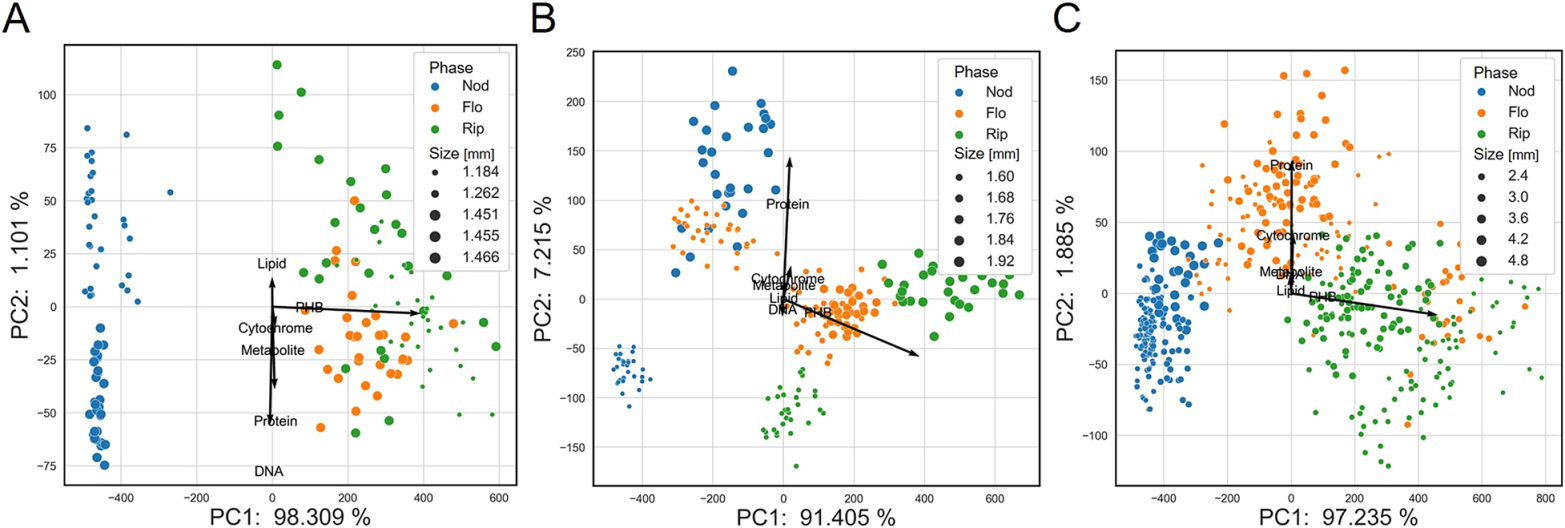
Figure 4. PCA score plots for each nodule size. A) G1. B) G2. C) G3. The plot size refers to the nodule size.

PCA was conducted to comprehensively examine the variations in bacteroid biomolecular profiles (Figure 4A, B, C). The data were divided by nodule size categories (G1, G2, and G3) because of the substantial differences in the ARA results among different-sized nodules, suggesting that biomolecular profiles evolve according to nodule size and, potentially, nodule age. The PCA score plots revealed distinct patterns in the biomolecular profiles. Bacteroids from different plant growth phases formed distinct clusters, primarily differentiated by PHB profiles, consistent with the observations in Figure 3. Within each plant growth phase, samples were also clustered according to nodule size. These size-dependent clusters were oriented perpendicularly to the plant growth phase-dependent clusters. The PHB content was the major contributor to the separation of plant growth phase clusters; however, the size-dependent clusters were characterized by variations in other molecular components.

### Prediction of nitrogen fixation activity from Raman spectral profiles

**Figure 5:**
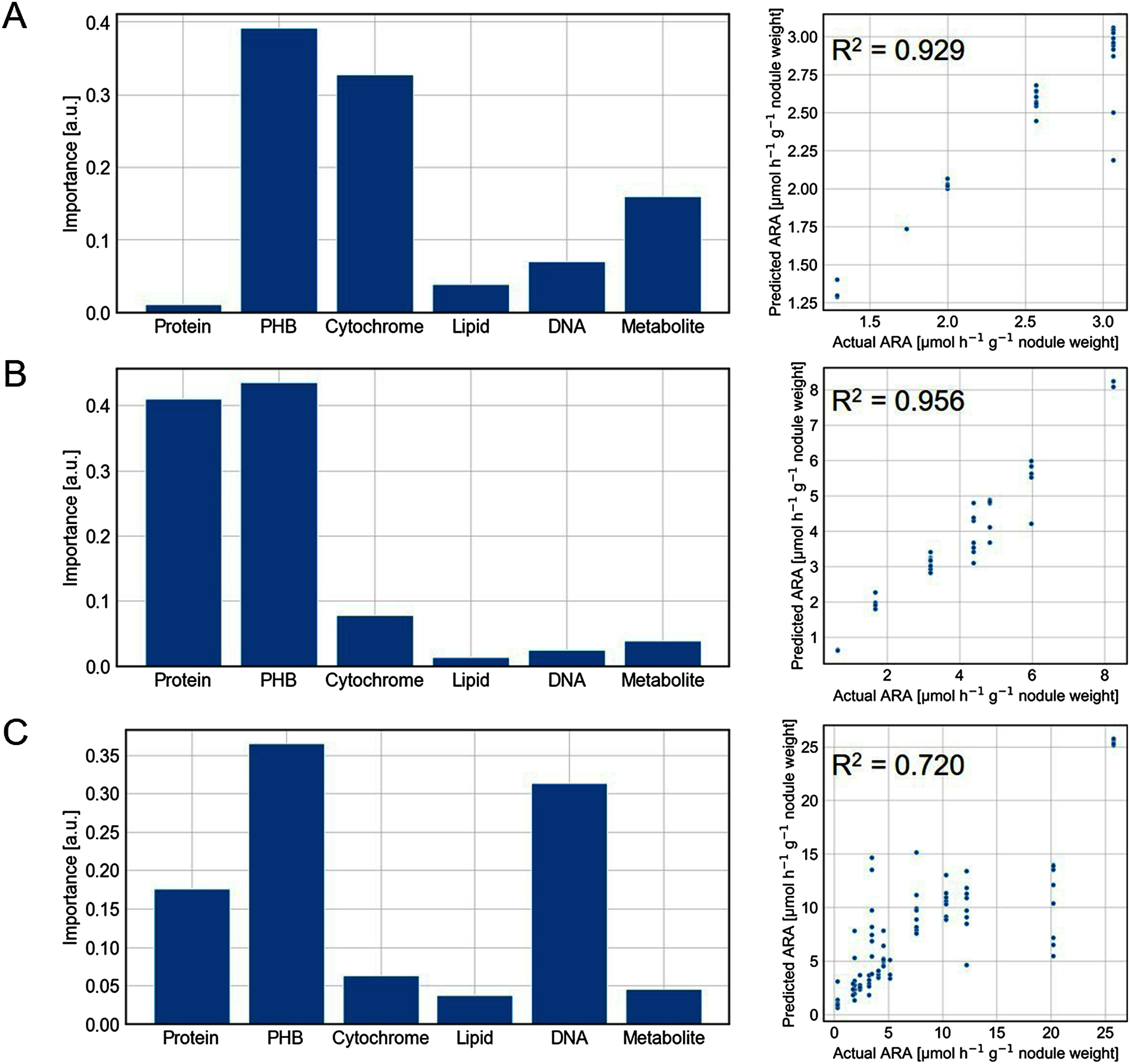
Figure 5. RFR used to predict the ARA results from Raman spectral constituents for each nodule size. A) G1. B) G2. C) G3. The bar plots refer to the importance of the constituents for the prediction. The score plots compare the actual and predicted ARA results of the test data, with the coefficient of determination R^2^.

RFR was performed to predict the symbiotic nitrogen fixation activity from bacteroid biomolecular profiles (Breiman 2001). The analysis was conducted for each nodule size group. Hyperparameter optimization was performed through k-fold cross-validation, using 75% of the dataset as training data. The optimized RFR model was then evaluated by predicting the ARA values, using the remaining 25% as test data. As a result, the constructed RFR model predicted the symbiotic nitrogen fixation (Figure 5A, B, C). The coefficients of determination (R^2^) exceeded 0.7 for all groups, with particularly high accuracy (R^2^>0.9) for the G1 and G2 nodules. The lower prediction accuracy for G3 nodules may be attributed to the broad size distribution within this group. While G1 and G2 groups exhibited narrow size distributions, G3 encompassed nodules ranging from 2 to 5 mm in diameter. This wide size range likely reflects heterogeneous nodule states, including developing and senescing nodules, potentially contributing to the reduced prediction accuracy. Notably, PHB emerged as a significant predictor for nitrogen fixation activity, suggesting its potentially crucial yet uncharacterized role in this process.

Another interesting finding was that the predictive model revealed distinct biomolecular contributions across nodule groups: cytochrome, protein, and DNA predominated in G1, G2, and G3 nodules, respectively (Figure 5A, B, C). Previous studies have documented quantitative and qualitative alterations in bacteroid cytochrome composition during symbiosis initiation, which establishes a microaerobic intracellular environment (Appleby 1984). This low-oxygen condition facilitates the utilization of plant-derived leghemoglobin. This suggests that cytochrome levels could serve as effective predictors of symbiotic nitrogen fixation activity within developing nodules. In G2 nodules, protein emerged as the second most significant contributor (Figure 5B). While specific protein identification remains challenging, this significance likely reflects the elevated enzymatic activities associated with symbiotic nitrogen fixation. DNA was highly important in G3 nodules (Figure 5C). These larger nodules are likely to approach senescence, characterized by a decreased nutrient supply from plant shoots and reduced cell division frequency (Kazmierczak et al. 2020). These results may indicate a correlation between bacteroid cell cycle regulation and symbiotic nitrogen fixation efficiency. The differential contributions of these biomolecules to symbiotic nitrogen fixation across nodule groups appear to reflect distinct molecular mechanisms during legume-bacteria symbiosis development. The observed difference might be due to that some nodules growing larger and containing more bacteroids.

The findings suggest that PHB plays a crucial role in symbiotic nitrogen fixation. While PHB has been extensively studied as a biodegradable biopolymer with potential applications in eco-friendly materials (Ratcliff et al. 2008; Trainer and Charles 2006), its function in soybean-rhizobia symbiotic nitrogen fixation remains largely unexplored. Nevertheless, several studies have demonstrated the impact of symbiont-produced PHB on host plant phenotypes. The inoculation with *phbC*-mutated *Sinorhizobium meliloti* altered *Medicago*’s phenotype, affecting shoot dry weight, nodule development, and acetylene reduction activity (Wang et al. 2007). *Phaseolus vulgaris* plants inoculated with PHB-nonproducing *Rhizobium etli* exhibited a reduced nitrogen content (Oono et al. 2021). The root mass of *Setaria viridis* was decreased by the inoculation with PHB-negative *Herbaspirillum seropedicae* (Silveira Alves et al. 2019). A plausible mechanism for PHB’s contribution to symbiotic nitrogen fixation involves its role as an energy reservoir for bacteroids. Although plant-derived carbon source is already known to be utilized for bacteroids survival and nitrogen fixation, the results indicate the involvement of intracellular PHB to symbiotic nitrogen fixation possibly as an additional energy source. Further investigation of the underlying mechanisms could provide insights into symbiotic nitrogen fixation, potentially leading to improved strategies for enhancing crop productivity in sustainable agriculture.

## Conclusion

In this study, we measured the symbiotic nitrogen fixation activity in soybean nodules of different growth stages and sizes. By leveraging single-cell Raman spectroscopy, we were able to analyze the biomolecular profiles of bacteroids within the nodules at an unprecedented level of detail and examine their correlations. Raman microspectroscopic measurements enabled the identification of essential cellular components, including proteins, cytochromes, and DNA, as well as the PHB biopolymer, which appeared as granules. This detailed profiling revealed that the amount of PHB fluctuated with plant growth. Furthermore, PCA score plots showed clear differences in biomolecular profiles depending on nodule conditions, with PHB serving as a key indicator of growth stages. Using random forest regression, we identified biomolecules significantly contributing to symbiotic nitrogen fixation, achieving high-accuracy predictions of the actual ARA values. Among these, PHB played a critical role, underscoring its biological relevance to nitrogen fixation.

Single-cell Raman spectroscopy was instrumental in these findings, as it enabled high-resolution, label-free characterization of complex biological systems. Although further research is necessary to elucidate the molecular behavior of PHB in this context, this discovery highlights the potential of advanced analytical technologies like Raman spectroscopy to deepen our understanding of nitrogen fixation mechanisms. This, in turn, could pave the way for innovations aimed at enhancing crop yields and promoting sustainable agriculture.

## Abbreviations

ARA: acetylene reduction assay
ASV: amplicon sequence variant
MCR-ALS: multivariate curve resolution-alternating least squares
OTU: operational taxonomic unit
PCA: principal component analysis
PHB: polyhydroxybutyrate
RFR: random forest regression

## Description of supplementary files

Supplementary Figure S1. Protocol for preparing the nodule extract for single-cell Raman measurement of bacteroids.

Supplementary Figure S2. The cross section of nodules taken at plant growth stage.

Supplementary Figure S3. Bacterial diversity analysis using the 16S rRNA gene sequencing of bacteroids extracted from nodules.

Supplementary Table S1. Row ARA values measured for the nodules at each growth stage.

Supplementary Table S2. The number of biological replicates for single cell Raman spectroscopic study.
