# Supplementary material for: Single-cell Raman spectroscopic analysis of bacteroids in soybean nodules to observe the relationship between biomolecular constituents and symbiotic nitrogen fixation activity
Source: Plant Biotechnol (Tokyo). 2025 Sep 25;42(3):335–43. doi: 10.5511/plantbiotechnology.25.0414a (PMC12573639; doi:10.5511/plantbiotechnology.25.0414a)
Supplement: Supplementary Data [file plantbiotechnology-42-3-25.0414a_s001.pdf]

Supporting Information

Single-cell Raman spectroscopic analysis of bacteroids in soybean nodules to observe the relationship between biomolecular constituents and symbiotic nitrogen fixation activity

Shunnosuke Suwa[1,2], Masahiro Ando[2,3], Kohki Kashiwagi[4], Takuma Kyotani[4], Kento Hasegawa[2,4], Habibi Safiullah[5], Masako Kifushi[2,4], Yohei Nishikawa[2,3,6], Toyoaki Anai[7], Naoko Ohkama-Ohtsu[8], Haruko Takeyama[2,3,4,9]

[1]Department of Advanced Science and Engineering, Graduate School of Advanced Science and Engineering, Waseda University;

[2]AIST-Waseda University Computational Bio Big Data Open Innovation Laboratory (CBBD-OIL);

[3]Research Organization for Nano and Life Innovation, Waseda University;

[4]Department of Life Science and Medical Bioscience, Graduate School of Advanced Science and Engineering, Waseda University;

[5]Faculty of Agriculture, Tokyo University of Agriculture and Technology;

[6]Biomanufacturing and Process Research Center (BPRC), National Institute of Advanced Industrial Science and Technology (AIST);

[7]Faculty of Agriculture, Kyushu University;

[8]Institute of Agriculture, Tokyo University of Agriculture and Technology;

[9]Institute for Advanced Research of Biosystem Dynamics, Waseda Research Institute for Science and Engineering, Waseda University;

*Corresponding author:

Haruko Takeyama (haruko-takeyama@waseda.jp)

Masahiro Ando (mando@aoni.waseda.jp)

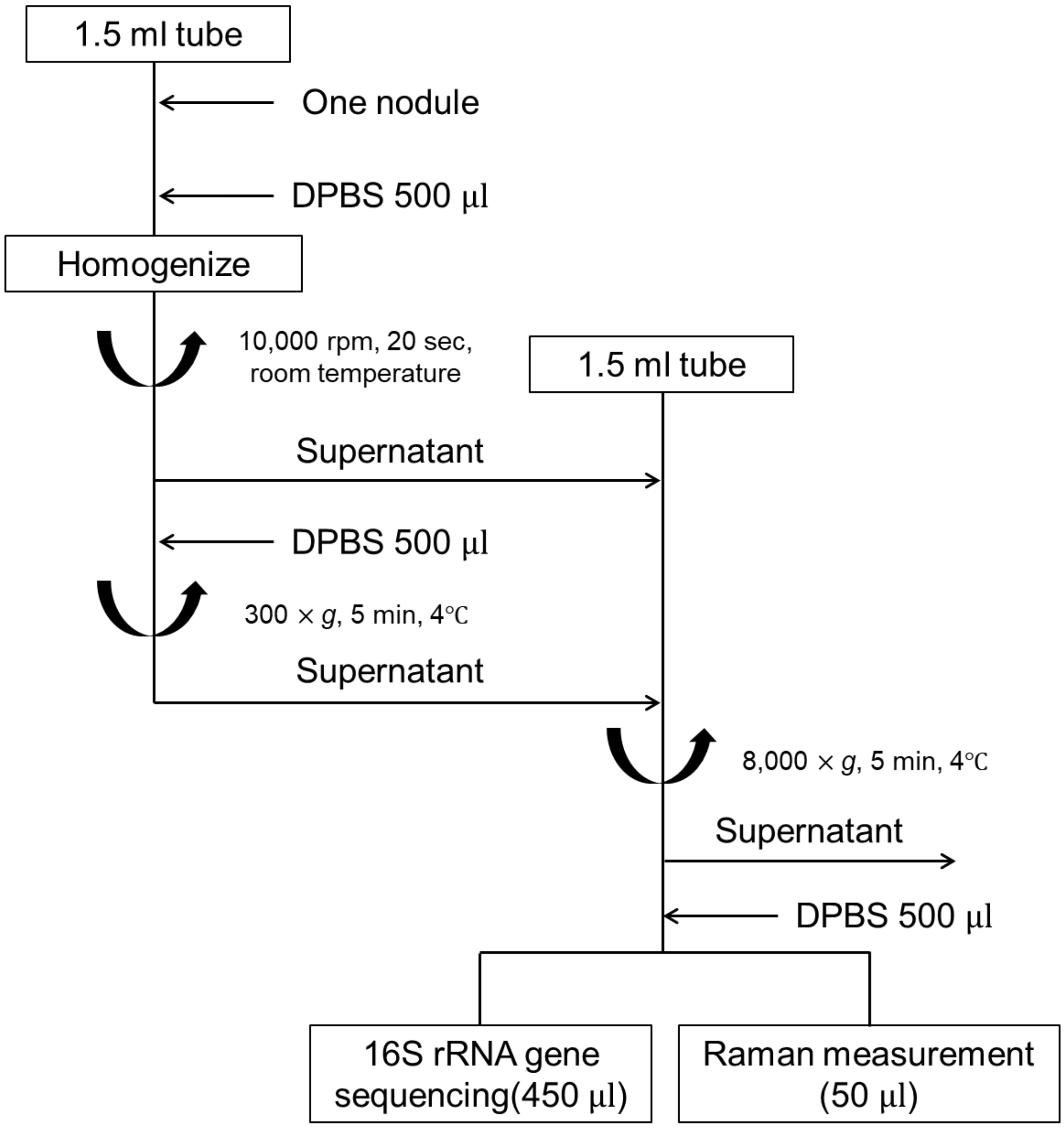

Supplementary Figure S1. Protocol for preparing the nodule extract for single-cell Raman measurement of bacteroids. First, the nodule was crushed in a 1.5 ml tube in 500 μl containing DPBS, and the suspension was centrifuged (10,000 rpm, 20 sec, room temperature). Second, the supernatant containing bacteroid was taken to another tube, and the remaining precipitation was suspended by 500 μl DPBS. The suspension was then centrifuged again (300 × *g*, 5 min, 4°C) and the supernatant was added to the second tube to fully collect the bacteroid. The resultant supernatant was centrifuged (8,000 × *g*, 5 min, 4°C) to collect bacteroid, and the supernatant was removed. Finally, 500 μl DPBS was added to the suspension, and was used for both Raman spectroscopic study and 16S rRNA gene sequencing.

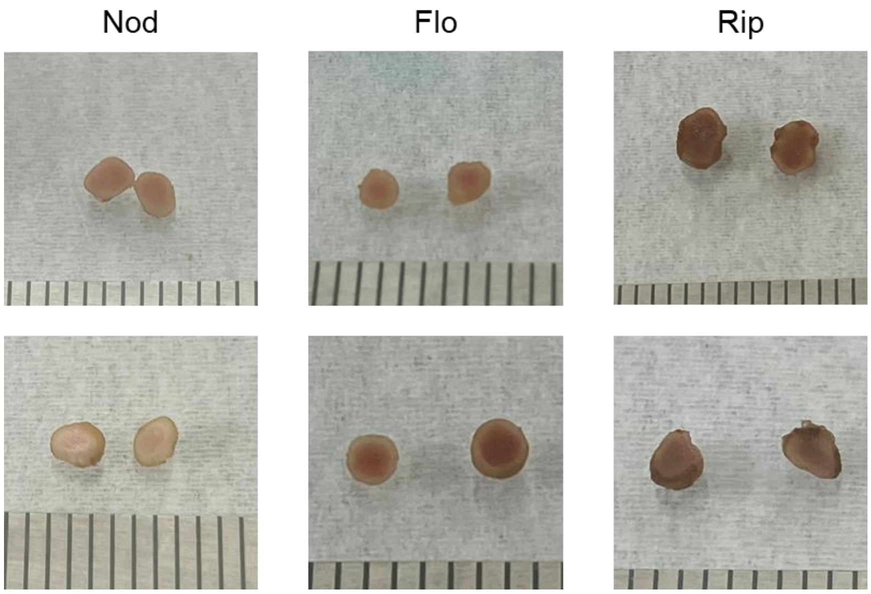

Supplementary Figure S2. The cross section of nodules taken at each plant growth stage.

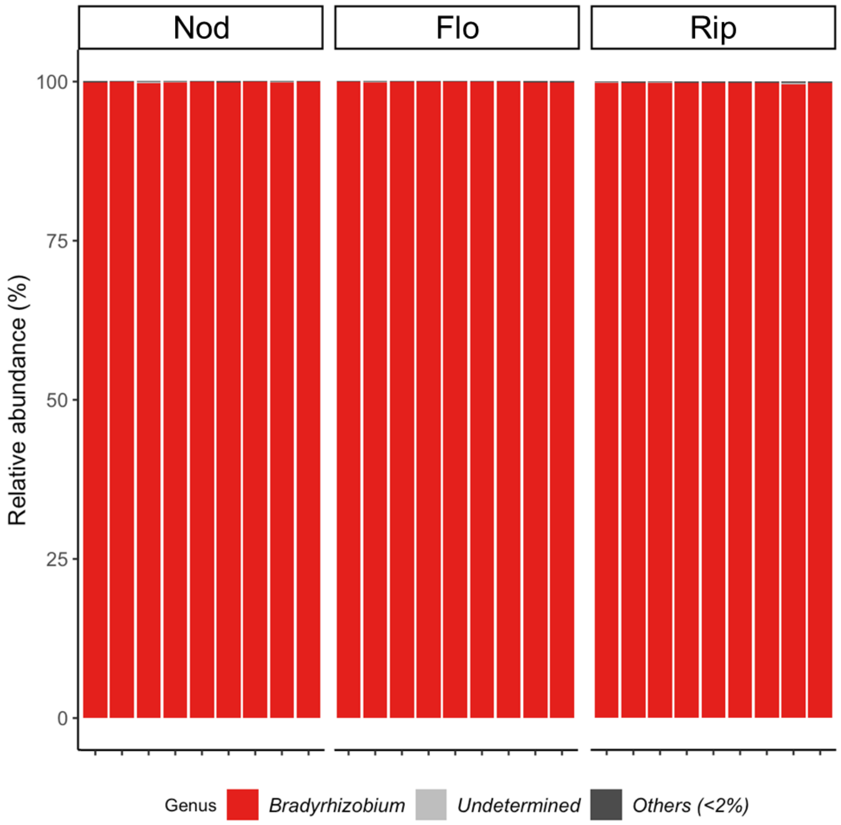

Supplementary Figure S3. Bacterial diversity analysis using the 16S rRNA gene sequencing of bacteroids extracted from nodules.

Supplementary Table S1. Row ARA values measured for the nodules at each growth stage.

	Nod: G1	Nod: G2	Nod: G3	Flo: G1	Flo: G2	Flo: G3	Rip: G1	Rip: G2	Rip: G3
Pot A	0.500477	5.9765222	25.754678	3.0660516	4.3831736	3.4620344	2.5695282	8.2407143	1.7264424
Pot B	1.7356392	4.532094	12.208375	1.6716669	1.8725533	4.0768045	1.9996711	2.3755058	0.3066794
Pot C	1.2890344	0.5490276	7.7991629	4.7330876	6.5751069	1.9672531	3.191904	5.1435032	3.2161481
Pot D	0.6316762	2.5356879	10.318117	4.8322662	7.5757524	20.20496	0.8484824	--- *	12.217586

Unit: µmol/hour/g nodule weight

* For the plant shoot in the pot C and D in Rip phase, each root did not have more than 15 nodules in G2. Therefore, ARA of Pot C Rip: G2 was measured by mixing nodules of pot C and D.

Supplementary Table S2. The number of biological replicates for single cell Raman spectroscopic study.

	Nod	Flo	Rip
G1	60	31	60
G2	59	93	61
G3	150	156	158
